# A Comparison of Affective Responses Between Time Efficient and Traditional Resistance Training

**DOI:** 10.3389/fpsyg.2022.912368

**Published:** 2022-06-16

**Authors:** Vidar Andersen, Marius Steiro Fimland, Vegard Moe Iversen, Helene Pedersen, Kristin Balberg, Maria Gåsvær, Katarina Rise, Tom Erik Jorung Solstad, Nicolay Stien, Atle Hole Saeterbakken

**Affiliations:** ^1^Faculty of Education, Arts and Sports, Western Norway University of Applied Sciences, Sogndal, Norway; ^2^Department of Neuromedicine and Movement Science, Faculty of Medicine and Health Sciences, Norwegian University of Science and Technology, Trondheim, Norway; ^3^Department of Public Health and Nursing, Faculty of Medicine and Health Sciences, Norwegian, Norwegian University of Science and Technology, Trondheim, Norway

**Keywords:** RPE, RPD, sPDF, EES, feeling

## Abstract

The aim of the study was to compare the acute effects of traditional resistance training and superset training on training duration, training volume and different perceptive measures. Twenty-nine resistance-trained participants (27 ± 7 years, 173 ± 9 cm, and 70 ± 14 kg) performed a whole-body workout (i) traditionally and (ii) as supersets of exercises targeting different muscle groups, in a randomized-crossover design. Each session was separated by 4–7 days, and consisted of eight exercises and three sets to failure. Training duration and number of repetitions lifted were recorded during the sessions. Rate of perceived exertion for effort (RPE), rate of perceived exertion for discomfort (RPD), session displeasure/pleasure (sPDF), and exercise enjoyment (EES) were measured 15 min after each session. Forty-eight hours after the final session participants reported which session they preferred. The superset session led to significantly higher values for RPE (1.3 points, *p* < 0.001, ES = 0.96) and RPD (1.0 points, *p* = 0.008, ES = 0.47) and tended to be higher for sPDF, i.e., more pleasurable, (*p* = 0.059, ES = 0.25) compared to the traditional session. There was no difference in EES (*p* = 0.661, ES = 0.05). The traditional session led to significantly increased training volume (4.2%, *p* = 0.011, ES = 0.34) and lasted 23 min (66%, *p* < 0.001, ES = 7.78) longer than the superset session. Eighteen of the participants preferred the superset session, while 11 preferred the traditional session. In conclusion, performing a whole-body workout as a superset session was more time-efficient, but reduced the training volume and was perceived with greater exertion for effort and discomfort than a traditional workout.

## Introduction

Improving muscular strength is associated with several health benefits and reduced risk of mortality (Jurca et al., 2004; Gale et al., 2007; Williams et al., 2007). Consequently, it is recommended to conduct resistance training 2–3 days per week for the major muscle groups (Garber et al., 2011). Still, most individuals do not follow these recommendations and lack of time is one of the most reported barriers (Hoare et al., 2017; Hurley et al., 2018). Therefore, finding time-efficient ways to perform resistance training is of great interest from both an individual and societal perspective.

A recently published review pinpointed several ways to reduce training duration when performing resistance training (Iversen et al., 2021). Iversen et al. recommended superset training as a method to substantially reduce training time. In contrast to traditional-set resistance training, where all sets of an exercise is completed before the next exercise, superset training could be defined as performing two or more exercises in succession with no, or limited, rest between them (Haff and Triplett, 2015). The exercises could target the same muscle groups, but this would primarily be relevant in a bodybuilding program with a training goal of producing high metabolic stress in a specific muscle group. However, if time efficiency is of an essence it is recommended to perform supersets that target different muscle groups. This allows for stimulating several muscle groups in shorter time, while not reducing the trained muscle groups recovery time, severely impairing performance in the next set, which would be the case if rest periods in traditional-set training were simply shortened (Iversen et al., 2021).

Performing the same work in a shorter period of time, have shown to increase the muscle fatigue (Paz et al., 2017) and blood lactate (Weakley et al., 2017) which could potentially limit the work performed in a session. Still, cross sectional studies comparing traditional resistance training to superset training where antagonists are paired, show no difference in training volume (Antunes et al., 2018) or even a higher volume for the superset training (Maia et al., 2014; Paz et al., 2017). This could be of importance since there seems to be a relationship between training volume and effects on muscle strength and hypertrophy (Schoenfeld et al., 2017; Grgic et al., 2018). Importantly, these studies (Maia et al., 2014; Paz et al., 2017; Antunes et al., 2018) are limited by only examining two exercises. When performing supersets over a whole training session, the accumulated fatigue could become more evident and result in reduced training volume.

How an activity is perceived, may be of importance for an individual regarding the choice to continue with the activity or not (Ekkekakis et al., 2005; Williams et al., 2008). As long as the rest interval are of similar length, Superset training sessions have less rest compared to traditional training sessions, due to fewer rest intervals, which in turn imposes more fatigue compared to traditional resistance training (Paz et al., 2017; Weakley et al., 2017). Consequently, it may be speculated that superset training is perceived as more exertive, discomforting and not as enjoyable as traditional training. Two cross-sectional studies have compared exertion between superset and traditional resistance training (Weakley et al., 2017, 2020). Both studies reported higher perceived exertion when conducting superset training than traditional training. However, both studies only included rating of perceived exertion (RPE). It has been recommended that RPE is accompanied by other measures, such as discomfort (Halperin and Emanuel, 2020).

We hypothesized that when performing a full body training program, superset training would lead to a reduced training volume in addition to greater levels of perceived discomfort, effort and displeasure compared to traditional resistance training among resistance trained individuals, and that superset training would be less enjoyable. Based on the hypothesis that the superset session led to higher levels of exertion and discomfort, and being less enjoyable, we expected that most participants would prefer traditional-set resistance training.

## Materials and Methods

### Study Design

**Figure 1:**
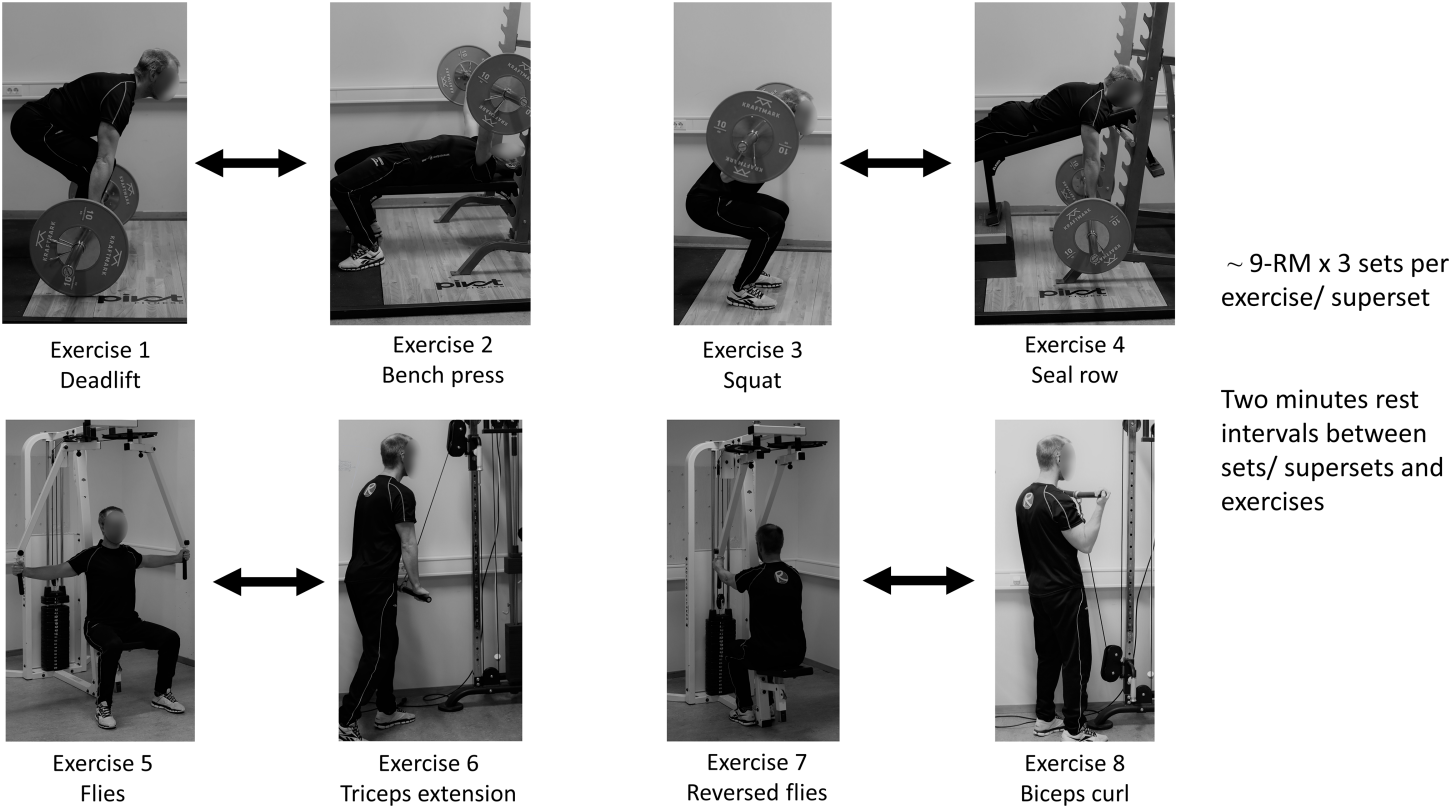
An overview of the traditional and the superset session. Bidirectional arrows indicate exercises performed in the same superset.

In this study, we used a within-subject, crossover design to compare the volume lifted and perceptive responses from a whole-body superset vs. a traditional-set resistance training session in resistance-trained individuals. The exercise program consisted of eight exercises (see Figure 1) with three sets each using ~ 9-RM loadings. The order of the sessions was randomized and counterbalanced, and all participants were required to partake in a familiarization session prior to two experimental sessions. Fifteen minutes after each session the participants were asked to report their session perception of effort, discomfort, pleasure/displeasure and enjoyment. In addition, training volume (number of repetitions lifted) and training duration were measured.

### Participants

Twenty-nine adults (15 females and 14 males) volunteered to participate in the study. They were recruited through posters, personal information, meetings, and social media. For anthropometrics see Table 1. The sample size was justified by performing *a priori* power analysis based on previous studies expecting a difference of 1.5 in RPE between the two sessions (Weakley et al., 2017, 2020), alpha level of 0.05 and power of 0.8. The inclusion criteria for participation were being over 18 years old, having more than 1 year experience with resistance training, being familiar with and able to perform the exercises with good technique, and not having any injuries which prohibited maximal exertion. All participants had experience with supersets, but not necessarily on a regular basis. The participants agreed to refrain from alcohol and resistance training 48 h in advance of each session. They were informed orally and in writing about the procedures, and provided a written consent before being enrolled in the study. The procedures were approved by the Norwegian Centre of Research Data (ref nr 424,466) and was conducted according to the University College’s ethical guidelines.

**Table 1:** Anthropometric data and self-reported 1-RM.

	All (*n* = 29)		Females (*n* = 15)		Males (*n* = 14)	
	Mean	SD	Mean	SD	Mean	SD
Age (years)	27.2	7.2	26.1	7.2	28.3	7.4
Body height (cm)	173.4	9.2	168.9	7.7	178.4	9.6
Body weight (kg)	70.2	14.0	62.1	7.0	79.9	13.9
Resistance training experience (years)	8.4	6.6	7.5	7.2	9.4	5.9
Self-reported 1-RM						
–Bench press (kg)	70.7	28.6	47.9	11.8	95.1	19.3
–Squat (kg)	91.5	32.0	68.9	12.5	115.7	28.5
–Deadlift (kg)	111.6	39.1	83.0	16.7	142.1	32.3

*RM, repetition maximum, SD, standard deviation*.

### Procedures

In the familiarization session anthropometrics were measured, the individual standardizations and load for the different exercises were determined (~ 9-RM) in addition to familiarizing the participants with the different scales. The intensity (9-RM) was chosen because it is in the middle of the range (6–12-RM) recommended for resistance-trained individuals (Garber et al., 2011). Since the participants were resistance trained and familiar with most of the exercises, they estimated their 9-RM in each exercise. If they were unsure, they performed sets with progressive loading in the specific exercise until they could report a specific load. Importantly, the same loads were used in both sessions. The scales were presented to the participants as a measure of the participants’ subjective experience of the sessions in turn of effort, discomfort, pleasure/displeasure, and enjoyment. Further, each scale was presented as in the experimental session (see below under measurements) and the participants were told that they should answer with the value representing their subjective assessment for that specific affection.

The two experimental sessions were conducted in a randomized order with 2–5 days between the sessions. In the beginning of each session, participants conducted a standardized warm-up consisting of two sets of deadlift, bench press, squat, and seal row. The first set consisted of six reps at 50% of the self-reported 9-RM and the last set consisted of six reps at 80% of self-reported 9-RM. The rest interval between each set was 90 s. After the final warm-up set the participants had 2 min rest before the session started. Three sets were conducted for each exercise using the same self-reported 9-RM load. In the traditional session, each set in one exercise was completed before a rest interval (2 min) while in the superset session two consecutive exercises (one set from each) were conducted immediately after each other before a similar rest interval (2 min). Otherwise, the sessions were equal. The resistance training program was a full-body program, consisting of eight exercises (see Figure 1 for an order of exercises and overview of the sessions). Training duration and training volume, defined as number of repetitions, were recorded during both sessions. Fifteen minutes after each session, participants were asked how they perceived the session related to effort, discomfort, pleasure/displeasure, and enjoyment. Forty-eight hours after the last session, participants were asked which of the two sessions they would use as their regular training routine, and the main reason for that choice.

The participants were instructed to complete as many repetitions as they could (i.e., until failure) in each set. The repetitions had to be performed continuously throughout the set, with a self-selected but controlled tempo (e.g., no bouncing allowed). The same test leader was present in all sessions for each individual to control that the standardizations noted in the familiarization-session were used, and to ensure that the execution of repetitions was as identical as possible within the set and between the sessions. Furthermore, the test leader kept track of time used in each session, counted the repetitions in each set, observed that the sets were at or close to failure and presented the scales to the participants. If the test leader perceived that the sets were not performed at or close to failure, he was instructed to remind the participants to complete as many repetitions as they could in each set. Of note, this was not needed during the data collection. To avoid distractions and keep the settings as similar as possible between sessions, all sessions were conducted in a lab with only one participant and the test leader present at the time. Of note, the participants had a minimum of 1 year of resistance training experience (average 8.4 years) and most of them were familiar with the exercises.

### Measurements

How the participants perceived the two sessions was assessed through four different scales. None of the participants had any previous experience with the scales. The scales were shown to the participants in the same order as listed below, 15 min after completing the last set. The participants were instructed to consider the whole session when giving their answers. All scales were shown to the participants while the test leader read the question to them (also listed on top of the scales). The scales were translated from the original forms to Norwegian. Prior to the study, three of the authors (AHS, HP, and VA) translated the scales independently before comparing, discussing, and agreeing on the final versions. These versions were then translated back to English by a professional. The new English versions were then compared with the originals. In general, there were only minor differences between the versions, which were adjusted after mutual agreement.

The perception of exertion was differentiated into effort and discomfort (Steele et al., 2016). Effort was measured using The rating of perceived exertion for effort scale (RPE), while discomfort was measured using the rating of perceived exertion for discomfort scale (RPD) (Fisher and Steele, 2017). Both scales consist of 11-items and ranges from no effort/discomfort to maximal effort/discomfort. Based on recent recommendations (Halperin and Emanuel, 2020), the RPE scale was presented to the participants with the following phrase: “*How much of your perceived physical capacity out of your perceived maximum (10 being your maximum) did you invest to complete this workout?*.” The upper and lower limit were anchored by the following sentence “*0 can be described as sitting still during the whole session while 10 would be maximal effort using your maximal physical capacity throughout the whole session*.” The RPD scale was presented with the following phrase: “*Based on the completed session, how much discomfort did you feel? The scale ends at 10 which could be described as you could not imagine the sensations relating to physical activity being any more intense?”* (Steele et al., 2016). The upper and lower limit were anchored by the following sentence *“0 can be described as feeling no noticeable sensation relating to the training while 10 would be the most intense training related sensation you could imagine.”*

The perceived pleasure/displeasure with the session was measured using the session pleasure/displeasure feelings scale (sPDF). The scale is a bipolar 11-point scale stretching from −5 (very bad) to 5 (very good), where 0 is considered neutral. The sPDF scale was presented with the following phrase: “*How was your workout?*” (Ribeiro et al., 2019). *The upper and lower limit were anchored by the following sentence “-5 can be described as perceiving the session as one of the worst/least pleasurable training sessions you have ever conducted while 5 would be one of the best/most pleasurable training sessions you have ever conducted.”* How much the participants enjoyed the sessions was measured using the exercise enjoyment scale (EES). The scale range is 1 (not at all)–7 (extraordinary). The scale was presented with the following question “*How much did you enjoy the exercise session?*” (Schwartz et al., 2021). The upper and lower limit were anchored by the following sentence “1 can be described as perceiving the session as one of the least enjoyable training sessions you have ever conducted while seven would be one of the most enjoyable training sessions you have ever conducted.”

Forty-eight hours after the last experimental session, the participants were contacted by e-mail and asked the following questions “*If you had to choose one of the two training sessions as your regular training session, which would you prefer, and what is the main reason for this choice?*.” The participants answered by replying to the mail. The answers were aggregated and grouped based on the underlying theme of the explanation.

### Statistical Analysis

The statistical analyses were performed using SPSS (IBM Corp. Released 2020. IBM SPSS Statistics for Windows, Version 27.0. Armonk, NY: IBM Corp). For the ordinal variables (RPE, RPD, sPDF and EES) the Wilcoxon signed rank test was used to compare the data between the sessions. The data regarding training duration and training volume (number of reps) was checked and confirmed for normality by visual inspection. Paired t-tests were used to assess differences between the two sessions. All results are presented as means ± standard deviations and Cohen’s *d* effect size (ES). ES was calculated using the following equation: mean pre-mean post divided by the pooled standard deviations of the two. Effect size was interpreted as 0.2 < *d* < 0.5 small; 0.5 < *d* < 0.8 medium, *d* ≤ 0.8 large (Cohen, 1988). Statistical significance was accepted at *p* ≤ 0.05.

## Results

The total number of repetitions conducted in the traditional session was 4.2% greater than the superset session (227 ± 29 vs. 218 ± 26 repetitions, *p* = 0.011, ES = 0.34, Table 2) while the training duration was 66% longer (58 ± 3 vs. 35 ± 3 min, *p* < 0.001, ES = 7.78).

**Table 2:** Accumulated number of repetitions for three sets using ~ 9RM loading for each exercise.

	Deadlift		Bench press		Squat		Seal row		Flies		Triceps extension		Reversed flies		Biceps curl	
	Mean	SD	Mean	SD	Mean	SD	Mean	SD	Mean	SD	Mean	SD	Mean	SD	Mean	SD
Traditional Superset	30	6	27	4	26	5	30	7	26	5	33	6	31	6	29	5
	30	6	25	3	26	5	26	6	26	4	28	5	29	4	27	5

*SD, standard deviation*.

The RPE was in average rated 1.3 points higher (*Z* = −3.845, *p* < 0.001, ES = 0.96, Table 3) and the RPD 1.0 point higher (*Z* = −2.671, *p* = 0.008, ES = 0.47) in the superset compared to the traditional session. Although not significantly different, there was a statistical trend for perceiving the superset session more pleasurable than the traditional session (*Z* = −1.891, *p* = 0.059, ES = 0.25). There was no significant difference in EES between the different sessions (*Z* = −0.440, *p* = 0.661, ES = 0.05).

**Table 3:** Perceptive measures for the traditional and superset session.

	Traditional		Superset			
	Mean	SD	Mean	SD	*p*-value	Effect size
RPE (0–10)	6.6	1.5	7.9	1.2^*^	<0.001	0.96
RPD (0–10)	5.4	2.2	6.4	2.0^*^	0.008	0.47
sPDF (−5–5)	2.8	1.9	3.3	2.0	0.059	0.25
EES (1–7)	4.5	1.3	4.6	1.3	0.661	0.05

* *, significantly different from traditional *p* < 0.05*.

*RPE, rate of perceived exertion effort, RPD, rate of perceived exertion discomfort, sPDF, session pleasure/displeasure, EES, exercise enjoyment*.

When asked which session they would prefer as their regular training session, 18 preferred the superset session while 11 preferred the traditional session.

## Discussion

The aim of this study was to compare the acute effects of supersets versus traditional-set resistance training on training volume and different perceptive measures. In accordance with our hypothesis the results showed that among resistance trained individuals, a whole-body superset training session that was considerably shorter than a traditional-set training session (35 vs. 58 min), led to 4% lower training volume and resulted in greater perceptions of discomfort and effort when compared to traditional resistance training. The superset session tended to be more pleasurable, but there were no differences in enjoyment. When asked what they would prefer in regular training, 18 participants answered supersets and 11 traditional sets.

There have been some previous studies comparing training volume and perceptive responses between traditional and superset resistance training (Maia et al., 2014; Paz et al., 2017; Weakley et al., 2017, 2020; Antunes et al., 2018). Interestingly, previous studies reported that training volume was similar (Antunes et al., 2018) or even increased (Maia et al., 2014; Paz et al., 2017) when conducting two exercises in a superset compared to separately. Importantly, these studies only used two exercises (1–3 sets per exercise) focusing on less muscle mass i.e., upper body (Paz et al., 2017) and single joint exercises (Maia et al., 2014; Antunes et al., 2018). Therefore, it appears that the decrease in performance during superset training first becomes apparent when the session includes multiple exercises and/or sets. This speculation is strengthened when comparing the training volume for the first two exercises in our study. Here, we did not find any difference in number of repetitions lifted between the two sessions (difference; 1.4 repetitions, *p* = 0.249). To the best of our knowledge, no previous study has compared perceived discomfort between traditional resistance training and superset training, but two studies have reported effort (Weakley et al., 2017, 2020). In agreement with our findings both studies reported greater RPE after completing two (Weakley et al., 2020) or six exercises (Weakley et al., 2017) in a superset session compared to a traditional session.

The reduction in training volume could be explained by increased fatigue in the superset session. Previous studies have shown that performing two exercises in a superset increases neuromuscular fatigue (Paz et al., 2017) and metabolic (e.g., increased lactate) and endocrine (e.g., cortisol) stress responses (Weakley et al., 2017). This increased stress may also explain the difference in effort and discomfort. It has been argued that performing sets until fatigue should yield a similar response in RPE (Fisher and Steele, 2017), however, in our study the participants were asked to consider the session as a whole and not after task failure in one set. Consequently, the difference in rating between the sessions may be explained by other factors than task-failure in each set. Of note, although effort and discomfort are different perceptions, they are reported to be related (Steele et al., 2016). Therefore, the increased perception of effort and discomfort could, at least partly, explain each other. Finally, the lack of difference in enjoyment could also be explained by our population. The fact that the participants were resistance trained with an average of 8 years of training experience indicate that they in general find enjoyment in performing resistance training.

The present study has some limitations that must be addressed. First, the participants in the study were resistance trained and the findings cannot necessarily be generalized to other populations, such as elite athletes or untrained individuals. Further, although all participants were familiar with supersets in their training routine, they did not necessarily use it on a regular basis. Therefore, it may be possible that their rating on the different perception scales would have been different if they had been using supersets more often. Although the different scales were presented to the participants in the familiarization session, they were not familiar with them prior to the study. The scoring might have been different if they had had more experience with the scales. Importantly, the order of the sessions was randomized so a potential familiarization effect should be similar for both sessions. Also, the measures were only assessed after the sessions. It has been shown that people are more positive toward training after the exercise (affective rebound effect; Ekkekakis et al., 2011). Therefore, the perception of the sessions may have changed throughout the sessions.

The intensity used in the sessions (9-RM) was subjectively reported by the participants. Therefore, it may be that the intensity was not the actual 9-RM. However, the reported intensity was close to the number of repetitions they were able to perform in the 9-RM testing, and it is unlikely that the deviation between reported and actual loading effected the results. Importantly the same load was used in both sessions to allow for comparisons between the two sessions. Also, we did not measure any physiological parameters such as heart rate, lactate etc. Such information could have provided additional insights.

From a practical point of view, performing superset training seems like a viable training form for resistance-trained individuals. As long as the length of the rest intervals are the same, superset training is more time efficient and is perceived more strenuous than traditional resistance training. Importantly time-efficiency and the feeling of working hard were the two major reasons why most of the participants (62%) preferred the superset session over the traditional session. Although, the superset session reduced the training volume compared to the traditional session by 4.2% (ES = 0.34), supersets can still be considered time efficient as the duration of the session was considerably shorter. These findings may be of individual and societal interest, if they can encourage individuals who struggle to find time for resistance training.

In conclusion, among resistance trained individuals, a whole-body superset training session that was considerably shorter than a traditional-set training session (35 vs. 58 min), led to 4% lower training volume, but greater perceptions of effort and discomfort when compared to traditional resistance training. Still, the superset session tended to be more pleasurable, and was preferred by most individuals. These findings suggest that those who are concerned about time efficiency and motivated for higher levels of exertion should favor superset training.

## Data Availability Statement

The raw data supporting the conclusions of this article will be made available by the authors, without undue reservation.

## Ethics Statement

Ethical review and approval was not required for the study on human participants in accordance with the local legislation and institutional requirements. The patients/participants provided their written informed consent to participate in this study.

## Author Contributions

VA came up with the idea and wrote the first draft. All authors helped in developing the methodology. KB, MG, and KR collected the data. All authors contributed to the article and approved the submitted version.

## Conflict of Interest

The authors declare that the research was conducted in the absence of any commercial or financial relationships that could be construed as a potential conflict of interest.

## Publisher’s Note

All claims expressed in this article are solely those of the authors and do not necessarily represent those of their affiliated organizations, or those of the publisher, the editors and the reviewers. Any product that may be evaluated in this article, or claim that may be made by its manufacturer, is not guaranteed or endorsed by the publisher.

## References

[ref1] Antunes L. Bezerra E. S. Sakugawa R. L. Dal Pupo J. (2018). Effect of cadence on volume and myoelectric activity during agonist-antagonist paired sets (supersets) in the lower body. Sports Biomech. 17:502. doi: 10.1080/14763141.2017.1413130, PMID: 29370715

[ref2] Cohen J. (1988). Statistical power analysis for the behavioral sciences (2nd Edn.), Hillsdale, NJ: L. Erlbaum Associates.

[ref3] Ekkekakis P. Hall E. E. Petruzzello S. J. (2005). Variation and homogeneity in affective responses to physical activity of varying intensities: an alternative perspective on dose-response based on evolutionary considerations. J. Sports Sci. 23, 477–500. doi: 10.1080/02640410400021492, PMID: 16194996

[ref4] Ekkekakis P. Parfitt G. Petruzzello S. J. (2011). The pleasure and displeasure people feel when they exercise at different intensities: decennial update and progress towards a tripartite rationale for exercise intensity prescription. Sports Med. 41, 641–671. doi: 10.2165/11590680-000000000-00000, PMID: 21780850

[ref5] Fisher J. P. Steele J. (2017). Heavier and lighter load resistance training to momentary failure produce similar increases in strength with differing degrees of discomfort. Muscle Nerve 56, 797–803. doi: 10.1002/mus.25537, PMID: 28006852

[ref6] Gale C. R. Martyn C. N. Cooper C. Sayer A. A. (2007). Grip strength, body composition, and mortality. Int. J. Epidemiol. 36, 228–235. doi: 10.1093/ije/dyl224 17056604

[ref7] Garber C. E. Blissmer B. Deschenes M. R. Franklin B. A. Lamonte M. J. Lee I. M.. (2011). American College of Sports Medicine position stand. Quantity and quality of exercise for developing and maintaining cardiorespiratory, musculoskeletal, and neuromotor fitness in apparently healthy adults: guidance for prescribing exercise. Med. Sci. Sports Exerc. 43, 1334–1359. doi: 10.1249/MSS.0b013e318213fefb, PMID: 21694556

[ref8] Grgic J. Schoenfeld B. J. Davies T. B. Lazinica B. Krieger J. W. Pedisic Z. (2018). Effect of resistance training frequency on gains in muscular strength: A systematic review and Meta-analysis. Sports Med. 48, 1207–1220. doi: 10.1007/s40279-018-0872-x, PMID: 29470825

[ref9] Haff G. G. Triplett N. T. (2015). “Essentials of strength training and conditioning 4th edition,” in Human Kinetics. Champaign, Illinois.

[ref10] Halperin I. Emanuel A. (2020). Rating of perceived effort: methodological concerns and future directions. Sports Med. 50, 679–687. doi: 10.1007/s40279-019-01229-z, PMID: 31745731

[ref11] Hoare E. Stavreski B. Jennings G. Kingwell B. (2017). Exploring motivation and barriers to physical activity among active and inactive Australian adults. Sports (Basel) 5. doi: 10.3390/sports5030047 PMC5968958 29910407

[ref12] Hurley K. S. Flippin K. J. Blom L. C. Bolin J. E. Hoover D. L. Judge L. W. (2018). Practices, perceived benefits, and barriers to resistance training Among women enrolled in college. Int. J. Exerc. Sci. 11, 226–238. 29795737 10.70252/ZRMT3507 PMC5955292

[ref13] Iversen V. M. Norum M. Schoenfeld B. J. Fimland M. S. (2021). No time to lift? Designing time-efficient training programs for strength and hypertrophy: a narrative review. Sports Med. 51, 2079–2095. doi: 10.1007/s40279-021-01490-1, PMID: 34125411 PMC8449772

[ref14] Jurca R.. (2004). Associations of muscle strength and fitness with metabolic syndrome in men. Med. Sci. Sports Exerc. 36, 1301–1307. doi: 10.1249/01.MSS.0000135780.88930.A9, PMID: 15292736

[ref15] Maia M. F. Willardson J. M. Paz G. A. Miranda H. (2014). Effects of different rest intervals between antagonist paired sets on repetition performance and muscle activation. J. Strength Cond. Res. 28, 2529–2535. doi: 10.1519/JSC.0000000000000451, PMID: 25148302

[ref16] Paz G. A. Robbins D. W. de Oliveira C. G. Bottaro M. Miranda H. (2017). Volume load and neuromuscular fatigue during an acute bout of agonist-antagonist paired-set vs traditional-set training. J. Strength Cond. Res. 31, 2777–2784. doi: 10.1519/JSC.0000000000001059, PMID: 28933712

[ref17] Ribeiro A. S. Dos Santos E. D. Nunes J. P. Schoenfeld B. J. (2019). Acute effects of different training loads on affective responses in resistance-trained men. Int. J. Sports Med. 40, 850–855. doi: 10.1055/a-0997-6680, PMID: 31499564

[ref18] Schoenfeld B. J. Ogborn D. Krieger J. W. (2017). Dose-response relationship between weekly resistance training volume and increases in muscle mass: a systematic review and meta-analysis. J. Sports Sci. 35, 1073–1082. doi: 10.1080/02640414.2016.1210197, PMID: 27433992

[ref19] Schwartz H. Emanuel A. Rozen Samukas I. I. Halperin I. (2021). Exploring the acute affective responses to resistance training: a comparison of the predetermined and the estimated repetitions to failure approaches. PLoS One 16:e0256231. doi: 10.1371/journal.pone.0256231, PMID: 34407124 PMC8372906

[ref20] Steele J. Fisher J. McKinnon S. McKinnon P. (2016). Differentiation between perceived effort and discomfort during resistance training in older adults: reliability of trainee ratings of effort and discomfort, and reliability and validity of trainer ratings of trainee effort. J. Trainol. 6, 1–8. doi: 10.17338/trainology.6.1_1

[ref21] Weakley J. J. S. Till K. Read D. B. Phibbs P. J. Roe G. Darrall-Jones J.. (2020). The effects of superset configuration on kinetic, kinematic, and perceived exertion in the barbell bench press. J. Strength Cond. Res. 34, 65–72. doi: 10.1519/JSC.0000000000002179, PMID: 28796130

[ref22] Weakley J. J. S. Till K. Read D. B. Roe G. A. B. Darrall-Jones J. Phibbs P. J.. (2017). The effects of traditional, superset, and tri-set resistance training structures on perceived intensity and physiological responses. Eur. J. Appl. Physiol. 117, 1877–1889. doi: 10.1007/s00421-017-3680-3, PMID: 28698987 PMC5556132

[ref23] Williams D. M. Dunsiger S. Ciccolo J. T. Lewis B. A. Albrecht A. E. Marcus B. H. (2008). Acute affective response to a moderate-intensity exercise stimulus predicts physical activity participation 6 and 12 months later. Psychol. Sport Exerc. 9, 231–245. doi: 10.1016/j.psychsport.2007.04.002, PMID: 18496608 PMC2390920

[ref24] Williams M. A. Haskell W. L. Ades P. A. Amsterdam E. A. Bittner V. Franklin B. A.. (2007). Resistance exercise in individuals with and without cardiovascular disease: 2007 update: a scientific statement from the American Heart Association Council on Clinical Cardiology and Council on Nutrition, Physical Activity, and Metabolism. Circulation 116, 572–584. doi: 10.1161/CIRCULATIONAHA.107.185214, PMID: 17638929

